# Nuclear envelope, chromatin organizers, histones, and DNA: The many achilles heels exploited across cancers

**DOI:** 10.3389/fcell.2022.1068347

**Published:** 2022-12-16

**Authors:** A. K. Balaji, Santam Saha, Shruti Deshpande, Darshini Poola, Kundan Sengupta

**Affiliations:** Chromosome Biology Lab (CBL), Indian Institute of Science Education and Research, Pune, Maharashtra, India

**Keywords:** lamins, heterochromatin, genome organization, nuclear envelope, oncohistones, histone variants

## Abstract

In eukaryotic cells, the genome is organized in the form of chromatin composed of DNA and histones that organize and regulate gene expression. The dysregulation of chromatin remodeling, including the aberrant incorporation of histone variants and their consequent post-translational modifications, is prevalent across cancers. Additionally, nuclear envelope proteins are often deregulated in cancers, which impacts the 3D organization of the genome. Altered nuclear morphology, genome organization, and gene expression are defining features of cancers. With advances in single-cell sequencing, imaging technologies, and high-end data mining approaches, we are now at the forefront of designing appropriate small molecules to selectively inhibit the growth and proliferation of cancer cells in a genome- and epigenome-specific manner. Here, we review recent advances and the emerging significance of aberrations in nuclear envelope proteins, histone variants, and oncohistones in deregulating chromatin organization and gene expression in oncogenesis.

## 1 Introduction

**FIGURE 1:**
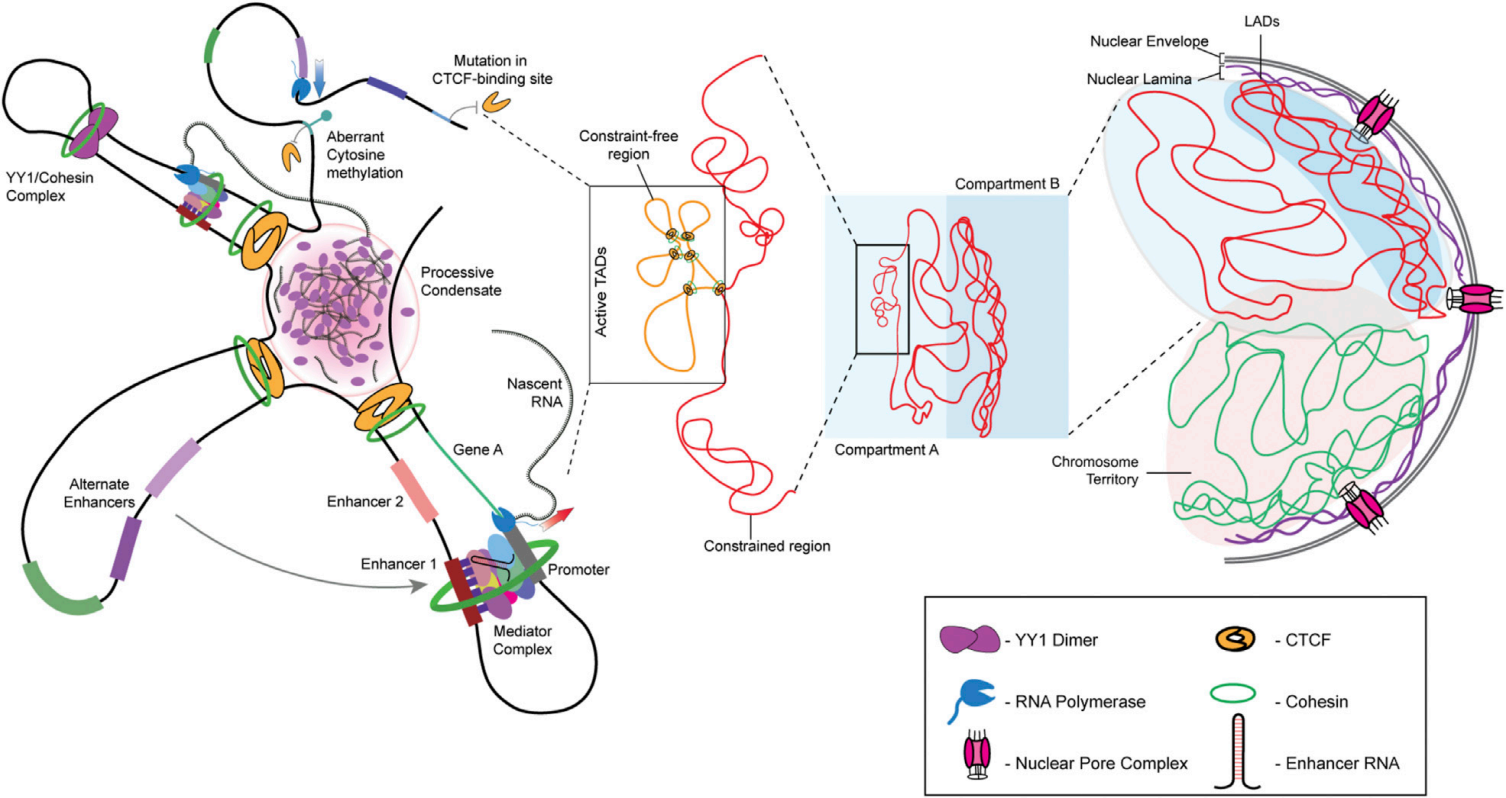
Hierarchy of genome organization. The involvement of lamins facilitates LAD organization at the nuclear periphery. Lamins organize the transcriptionally inactive B compartment of the TADs proximal to the nuclear envelope, while the transcriptionally active A compartment is maintained relatively toward the nuclear interior. The promoter-enhancer contacts are maintained by genome organizers such as cohesin, condensin, and CTCF. The presence of multiple such contacts within a TAD allows the formation of splicing condensates.

Each chromosome occupies a unique sub-volume in the interphase nucleus, referred to as a chromosome territory (Cremer and Cremer, 2001). A chromosome territory encompasses intra- and inter-chromatin interactions, further fine-tuned by histone modifications. Analyses of chromatin interactions have revealed the organization of chromatin into two distinct compartments—A and B. Compartment A is composed of gene-rich, open chromatin localized toward the nuclear interior. In contrast, compartment B is gene-poor, has a compact conformation, and is localized toward the nuclear periphery. Closer inspection using variants of chromosome conformation capture assays, such as 3C, 4C, and Hi-C, reveals that the 3D genome architecture of a nucleus is intricately organized into Topologically Associating Domains (TADs), where stretches of chromatin physically interact in a regulated manner to modulate gene expression within the TAD (Figure 1) (Szabo et al., 2019). The loop extrusion model of chromatin organization forms the basis of TAD-mediated genome organization, where chromatin loops are extruded by chromatin organizers and cohesin complexes and are delimited by CCCTC-binding factor (CTCF), another chromatin organizer (Fudenberg et al., 2016; Nuebler et al., 2018). TADs organize looping-in of sequences ∼1 Mb (in mammals) apart within close proximity, enabling enhancer-promoter contacts for the spatiotemporal regulation of gene expression (Chetverina et al., 2017). Interestingly, chromatin stretches with the same type of histone modifications show a propensity to interact and compartmentalize in the 3D space of the nucleus, thus revealing CTCF-cohesin-independent chromatin folding mechanisms. For instance, H3K27me3 histone modifications function as a signal for long-range chromatin interactions during hematopoietic stem cell differentiation (Zhang X et al., 2020). Notably, in addition to CTCF, genome organizers such as cohesin and condensin are required for the recruitment of transcription cofactors. Cohesin and CTCF function as boundary elements that collectively maintain genome architecture, which is prudently rigid and guardedly dynamic (Phillips-Cremins et al., 2013). Furthermore, chromatin remodelers alter local chromatin dynamics in response to cell signaling events. The maintenance of TADs is critical since disruption of TAD organization is associated with developmental diseases and cancers (Lupiáñez et al., 2015; Akdemir et al., 2020). In cancers, the aberrant activation of cell signaling pathways relays erratic signals to the nucleus, which alters chromatin organization and transcriptional outputs of the cell. It remains to be examined how chromatin and its organizers respond to aberrant oncogenic signaling in cancer cells.

The double-membraned nucleus functions as the primary protector of the genome. In metazoans, the nucleus not only harbors the genome but also works in tandem with the differentially-compacted chromatin to regulate its tissue-specific spatial and functional organization. The nuclear envelope comprises the nuclear lamina that maintains nuclear integrity and regulates gene expression and is interspersed with Nuclear Pore Complexes (NPCs), whose primary function is to regulate nuclear transport (Lin et al., 2018). Though the chromatin in contact with the nuclear lamina is frequently repressed, NPCs additionally contribute to the regulation of gene expression. Furthermore, owing to the role of NPCs in chromatin organization and function, Nucleoporins (Nups), the class of proteins that comprise the NPC, are also involved in regulating stemness and cell fate determination (D’Angelo et al., 2012). Interestingly, nucleoporins crosstalk with the chromatin organizer CTCF, which functions as a boundary element between TADs, while facilitating intra-TAD interaction. For instance, the nucleoporins Nup153 and Nup93, along with CTCF, regulate the transcriptional activity of the HOX gene cluster during early development and differentiation (Kadota et al., 2020; Labade et al., 2021).

In metazoans, type V intermediate filament proteins, the lamins, maintain the structural and functional integrity of the nucleus (Aebi et al., 1986; Gruenbaum and Foisner, 2015). The nuclear lamina is predominantly composed of two lamin sub-types—the A-type lamins that include lamins A and C (a spliced variant of lamin A), and the B-type lamins that comprise separately-encoded lamins B1 and B2 (in vertebrates). Each lamin sub-type harbors post-translational modifications (PTMs), exponentially increasing the functional diversity of lamins. Nuclear lamins regulate replication-dependent cell cycle progression, DNA damage repair, genome stability, and 3D organization of the genome (Moir et al., 2000; Bronshtein et al., 2015; Earle et al., 2020). Nuclear lamina interacts with stretches of chromatin that are in proximity to the nuclear periphery, referred to as Lamina-Associated Domains (LADs). LADs are typically repressed, barring exceptions where a subset of euchromatin interacts with lamin B1 and are categorized as euchromatic LADs (eLADs) (Guelen et al., 2008; Pascual-Reguant et al., 2018).

Chromatin in eukaryotes is organized as DNA wrapped around histone octamers, forming nucleosomes. Further, the linker histone H1 is incorporated with the nucleosomes constituting the fundamental units of the chromatin fiber—the chromatosomes (Zhang and Li, 2017). Histones are among the most widely modified proteins, and each modification has the unique ability to regulate gene expression (Turner, 1993; Millán-Zambrano et al., 2022). Actively transcribing genomic regions localized away from the nuclear periphery are often associated with active histone marks such as H3K4me3, H3K9ac, and H3K27ac, deposited by histone remodelers such as KMT2, CBP, and p300, respectively (Ogryzko et al., 1996; Rao and Dou, 2015). On the other hand, histone modifications such as H3K9me2/3, H3K27me3, and H4K20me1 are associated with transcriptional repression. The combination of active and inactive marks fine-tunes transcriptional output (Kimura, 2013; Talbert and Henikoff, 2021). Of note, repressive histone marks such as H3K9me2/3 and H3K27me3, deposited by INM-interacting histone-remodeling complexes such as SUV39H1/2 and the Polycomb Repressor Complex 2 (PRC2) complex, are often enriched on LADs (Shumaker et al., 2006; Cesarini et al., 2015; Harr et al., 2015). These histone-remodeling complexes interact with lamins and maintain the associated chromatin in a state of repression (Marullo et al., 2016; Salvarani et al., 2019; Bianchi et al., 2020; Siegenfeld et al., 2022).

Cancer cells exhibit a remarkable interplay between aberrant genome organization and deregulated transcription. The cancer genome harbors mutations in both coding and non-coding regions, selectively providing tumorigenic cells with a proliferative advantage to outcompete normal cells (Moreno and Basler, 2004; Pon and Marra, 2015; Bailey et al., 2021). For instance, incorporating non-canonical histone variants alters nucleosome stability, often resulting in altered replication and transcription (Bönisch et al., 2012; Henikoff and Smith, 2015; Buschbeck and Hake, 2017). In solid tumors, mutant chromatin remodelers differentially recruit histone modifiers that confer chemoresistance (Drosos et al., 2022). These cancer-associated mutant histones are referred to as oncohistones, which are now emerging as a class of prominent biomarkers of cancers (Bočkaj et al., 2021). In this review, we address the molecular and mechanistic underpinnings of nuclear envelope factors, their crosstalk with chromatin, and their pivotal role in cancer initiation and progression.

## 2 Nuclear envelope and lamins

**FIGURE 2:**
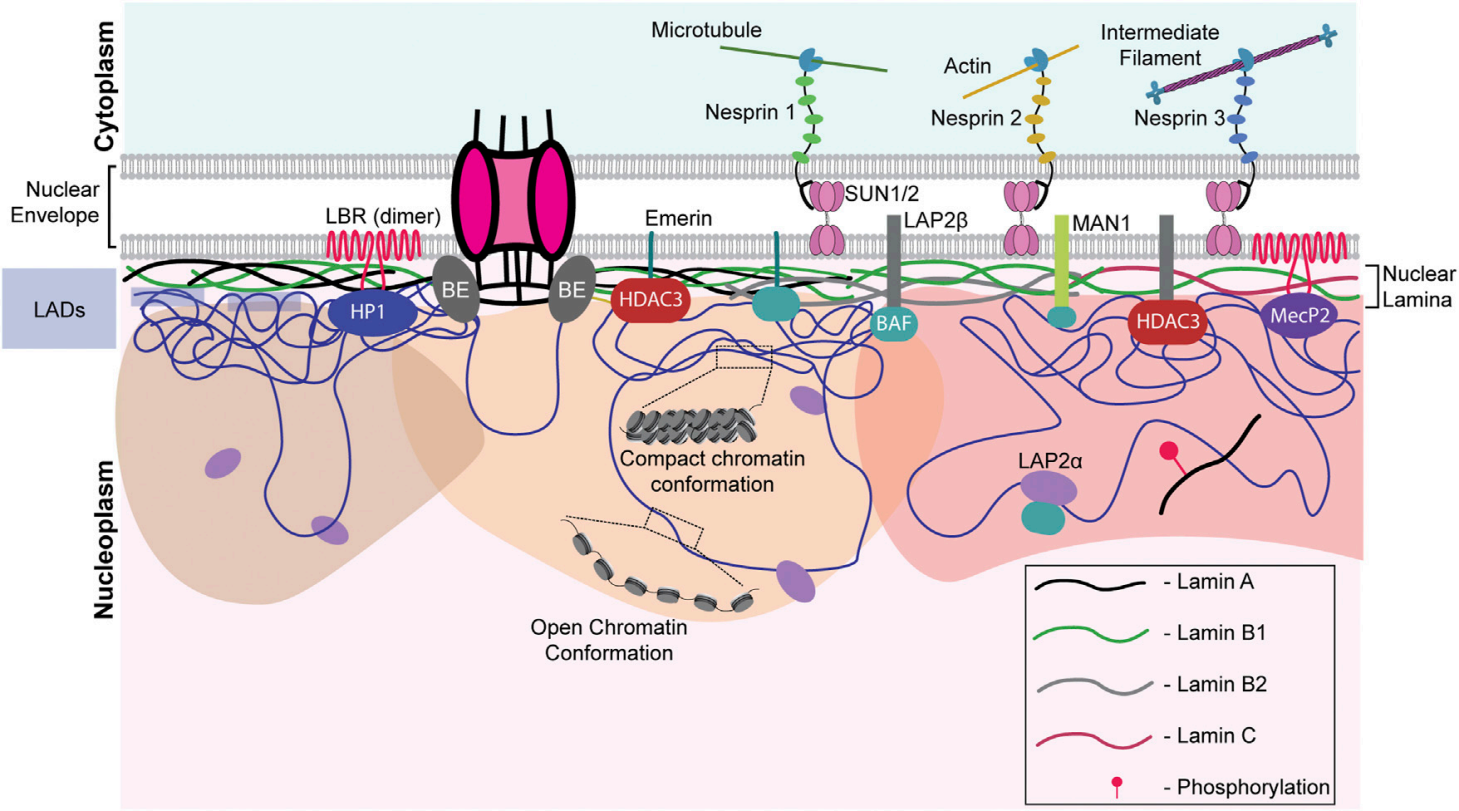
Nuclear envelope architecture and its role in genome organization. A schematic model showing the INM proteome involved in maintaining genome organization. Nesprins communicate external mechanical cues to the nucleus *via* the SUN1/2 complex. The regions of the genome contacting the nuclear lamina, the LADs, are maintained in a heterochromatic state. Phosphorylated lamin A binds to active enhancers in the nuclear interior. LAP2α interacts with intra-nuclear lamin A/C and could be regulating its functioning. Chromatin remodelers such as HDACs and sirtuins are often associated with the INM proteins such as emerin, MAN1, LAP2β, lamins, and LBR. NPCs are also associated with chromatin. LAP2α - lamina Associated Peptide 2α, LBR - lamin B receptor, BE - Barrier Element, HP1—Heterochromatin Protein 1, HDAC—histone deacetylase, SUN1/2—Sad1/unc-84 protein-like 1/2 and MeCP2—methyl CpG-binding protein 2.

The nuclear envelope is a crucial barrier between the cytoplasm and the nucleus and functions as a protector of the genome. The nuclear envelope consists of an outer and inner nuclear membrane (ONM and INM, respectively) and NPCs. The INM is lined on the inner side by a protein meshwork referred to as the nuclear lamina, which is composed of type V intermediate filament proteins—the A- and B-type lamins. The A-type lamins, lamin A/C, are produced as two somatic isoforms of prelamin A by alternative mRNA splicing at exon 10 of the LMNA gene (Machiels et al., 1996). Lamin A bears a CaaX motif at its C-terminal end, which is farnesylated, in contrast to lamin C, which does not contain the CaaX motif or undergo farnesylation (Vorburger et al., 1989). Notably, mature lamin A is formed by the loss of farnesylation inside the nucleus. On the other hand, the predominant B-type lamins—lamins B1 and B2 are permanently-farnesylated products of two separate genes—LMNB1 and LMNB2. Lamins interact with chromatin, either directly or through transmembrane proteins of the INM—lamin B receptor (LBR), MAN1, and Emerin (Ye and Worman, 1996; Holaska and Wilson, 2007; Demmerle et al., 2012). Apart from biochemical cues, the nucleus directly perceives mechanical signals through the LINC complex, involving Nesprin and SUN proteins (Figure 2). Nesprins, which extend from the ONM to the perinuclear space, directly interact with cytoskeletal proteins such as vimentin, actin, and microtubules (Ketema et al., 2013; Gimpel et al., 2017; Li et al., 2021). Mechanical cues are subsequently propagated *via* the interaction with the SUN and KASH domain proteins (Starr and Fischer, 2005; Tzur et al., 2006). These lines of evidence suggest that the coordinated functioning of nuclear lamins, nucleoporins, and LINC complex factors is central to the functional organization of the nucleus and the genome.

Aberrations in nuclear morphology, such as invaginations, blebs, and micronuclei, serve as histological markers for grading tumor progression and often correlate with carcinogenesis. For instance, Haematoxylin-Eosin (HE) staining of papillary thyroid carcinoma cells and colon adenocarcinoma revealed enlarged nuclei with irregular morphology compared to normal cells with smaller and spherical nuclei (Fischer, 2020). Furthermore, nearly 90% of solid tumors are characterized by aneuploidy, which predominantly involves deletions and amplifications at the whole chromosomal and sub-chromosomal levels (Holland and Cleveland, 2012). CIN leads to transcriptional imbalances in a cell- and tissue-specific manner (Bakhoum et al., 2018; Benhra et al., 2018). Here, we focus on the mechanisms by which defects in the nuclear envelope manifest themselves, resulting in genomic instability and thereby contributing to cancer progression.

### 2.1 Role of aberrant nuclear envelope factors in cancers

The stability and integrity of LINC complex proteins and nuclear lamins are crucial for maintaining chromatin organization and genome stability, aberrations of which are associated with various cancers (Sur et al., 2014). For instance, immunohistochemistry showed decreased expression of LMNA and LMNB1 in 7/8 primary gastric cancers and 6/8 gastric cancers, respectively (Moss et al., 1999). In addition, nuclear envelope proteins regulate chromosomal stability as they participate in cell cycle progression, chromosome segregation, and nuclear envelope assembly post-mitosis (Dechat et al., 2007; Kuga et al., 2014; Dubińska-Magiera et al., 2019).

Remarkably, nuclear morphology plays a vital role in modulating cell fate in the continuum of cancer progression (Capo-Chichi et al., 2016; Smith et al., 2018; Fischer, 2020). In particular, the loss of emerin and lamin A show aberrations in nuclear morphology, accompanied by an increased aggressiveness of cancer cells (Reis-Sobreiro et al., 2018; Bell et al., 2022). Consistent with this finding, ovarian cancers show decreased emerin and lamin A/C levels, accompanied by a progressive destabilization of the nuclear envelope (Capo-chichi et al., 2011). Lamin A/C-emerin co-depletion alters chromatin mobility, suggestive of their role in the maintenance of genome organization and function (Ranade et al., 2019). Intriguingly, depletion of lamin A/C mislocalized emerin, resulting in altered nuclear morphology and increased invasiveness of DU145 prostate cancer cells (Kong et al., 2012; Reis-Sobreiro et al., 2018).

The nuclear lamina is composed of three lamin sub-types and interacts with the LINC complex genes, and this confers a molecular redundancy on lamin function, which counters abrupt alterations in the lamina—predominantly in response to external cues. For instance, keratinocytes and fibroblasts of the skin derived from lamin A/C-knockout mice showed prolonged expression of LBR as compared to wild-type cells (Solovei et al., 2013). Similar buffering mechanisms were uncovered in EMD- and LMNA-null mice during development. While LAP2α was upregulated in myogenic cells derived from LMNA^−/−^ mice, cells derived from EMD-null mice showed a compensatory increase in lamin A expression (Melcon et al., 2006). However, the mechanistic basis of the transcriptional feedback circuits between lamin A/C and the nuclear envelope factors remains to be examined in greater detail.

The nuclear lamina functions as a docking site for anchoring LADs enriched in heterochromatin. For example, LBR tethers heterochromatin to the nuclear envelope in actively proliferating cancer cells during the early stages of mammalian development, while lamin A/C is a chromatin anchor in differentiated cells (Solovei et al., 2013; Lukášová et al., 2017). It is interesting to note that the loss of both LBR and lamin A/C results in the inversion of chromatin with heterochromatin toward the nuclear interior (Solovei et al., 2013), reiterating the significance of the nuclear lamina in chromatin organization and function. Furthermore, lamin B1 loss significantly increases nuclear bleb formation, while the depletion of lamin A/C shows morphological aberrations such as nuclear atypia, in addition to aneuploidy and CIN (Lammerding et al., 2006; Capo-chichi et al., 2011). Furthermore, destabilization of the nuclear envelope shows enhanced nuclear blebbing and micronuclei formation, which contribute to chromosomal losses and aneuploidy (Capo-Chichi et al., 2016). In addition to A and B-type lamins, peripheral heterochromatin provides additional stiffness, and its deregulation foreseeably weakens the nuclear envelope, contributing to the formation of nuclear blebs (Stephens et al., 2018). In summary, a stable nuclear envelope composition is required for genome organization facilitated by the maintenance of nuclear integrity by reinforcing nuclear stiffness.

Interestingly, the loss of lamin B1 shows CIN and DNA damage by destabilizing key Homologous Recombination (HR) pathway proteins such as Rad51 in U2OS cells (Liu et al., 2015). Correspondingly, A-type lamins regulate HR through transcriptional co-regulation of RAD51 and BRCA1 while modulating Non-Homologous End Joining (NHEJ) through 53BP1 in breast cancer cells (Redwood et al., 2011). Lamin A also regulates DNA damage repair (DDR) *via* its direct interaction with Hsp90—a molecular chaperone involved in protein folding and stability in ovarian cancer cells (Wang et al., 2021). Furthermore, whether the differential stoichiometry of the A and B-type lamins modulates NHEJ or HR pathways to repair damaged DNA in a cell-type- and cancer-specific manner remains an open question.

### 2.2 Role of nucleoporins in genome organization and cancers

Nuclear pore complexes (NPCs) are ∼120 nm-wide structures in the nuclear envelope, which mediate selective transport in and out of the nucleus. In vertebrates, NPCs comprise nucleoporins (Nups), a group of ∼30 proteins, to form a ∼125MDa protein complex (Cronshaw et al., 2002; Cohen et al., 2012). In addition to nuclear transport, the NPCs regulate chromatin interaction and function (Zhou and Panté, 2010; Kadota et al., 2020). Further, Nups are classified into 1) on-pore Nups that are associated with the NPC and 2) off-pore Nups that exist both in the NPC and nucleoplasm. Nups that interact with and regulate essential genes in the genome include Nup93 (on-pore) and Nup153 (off-pore), which interact with super-enhancers, and function as major chromatin regulators (Baumann, 2016; Ibarra et al., 2016). Nup153 and Nup98, present near the nuclear basket of the NPC, communicate with a wide range of poised genes through interactions with CTCF (Pascual-Garcia et al., 2017). Certain on-pore Nups, such as Nup93, interact with and repress HOXA genes which are essential for early development (Labade et al., 2016). In the context of cancer progression, Nup93 facilitates metastasis by enhancing β-catenin import and upregulating EMT target genes, thus inducing epithelial-to-mesenchymal transition (EMT) in breast and hepatocellular cancers (Lin et al., 2022; Nataraj et al., 2022). The on-pore Nup210 interacts with SUN2 to regulate the expression of prometastatic mechanosensitive genes by impeding the spread of heterochromatin (Amin et al., 2021). Intriguingly, a non-canonical extranuclear function of the Nup107-160 complex is to stabilize bipolar spindle arrangement and prevent aneuploidy during each cell division, thus maintaining genome integrity (Orjalo et al., 2006).

Nups contribute to cancer progression by forming fusion proteins. For instance, the off-pore Nup98 is involved in multiple fusion proteins with transcriptional coactivators, histone methyltransferases, helicases, and in some instances, even orphan proteins (Wang et al., 2007; Yassin et al., 2010; Gough et al., 2011). During hematopoietic stem cell differentiation, HoxA7, HoxA9, and HoxA10 levels are upregulated, which progressively decrease as the hematopoietic cells differentiate further into various lineages. The Nup98 fusion protein alters gene expression resulting in Acute Myeloid Leukemia (AML). Intriguingly, in Nup98-NSD1 fusion protein-mediated AML, the fusion protein is recruited to the HoxA7, HoxA9, and HoxA10 gene loci. The FG-repeat (originating from Nup98) of the fusion protein interacts with HAT CBP/p300, leading to overexpression of the aforementioned Hox genes, contributing to AML. Nup98-PHD fusion proteins also promote AML progression *via* a similar mechanism (Wang et al., 2007, 2009). Thus, the aberrant localization and expression of Nups impact genome organization, contributing to oncogenesis. Taken together, the cellular machinery effectively copes with aberrant gene expression—a major target for future therapeutic approaches.

### 2.3 Lamins modulate copy number alterations in cancers

**FIGURE 3:**
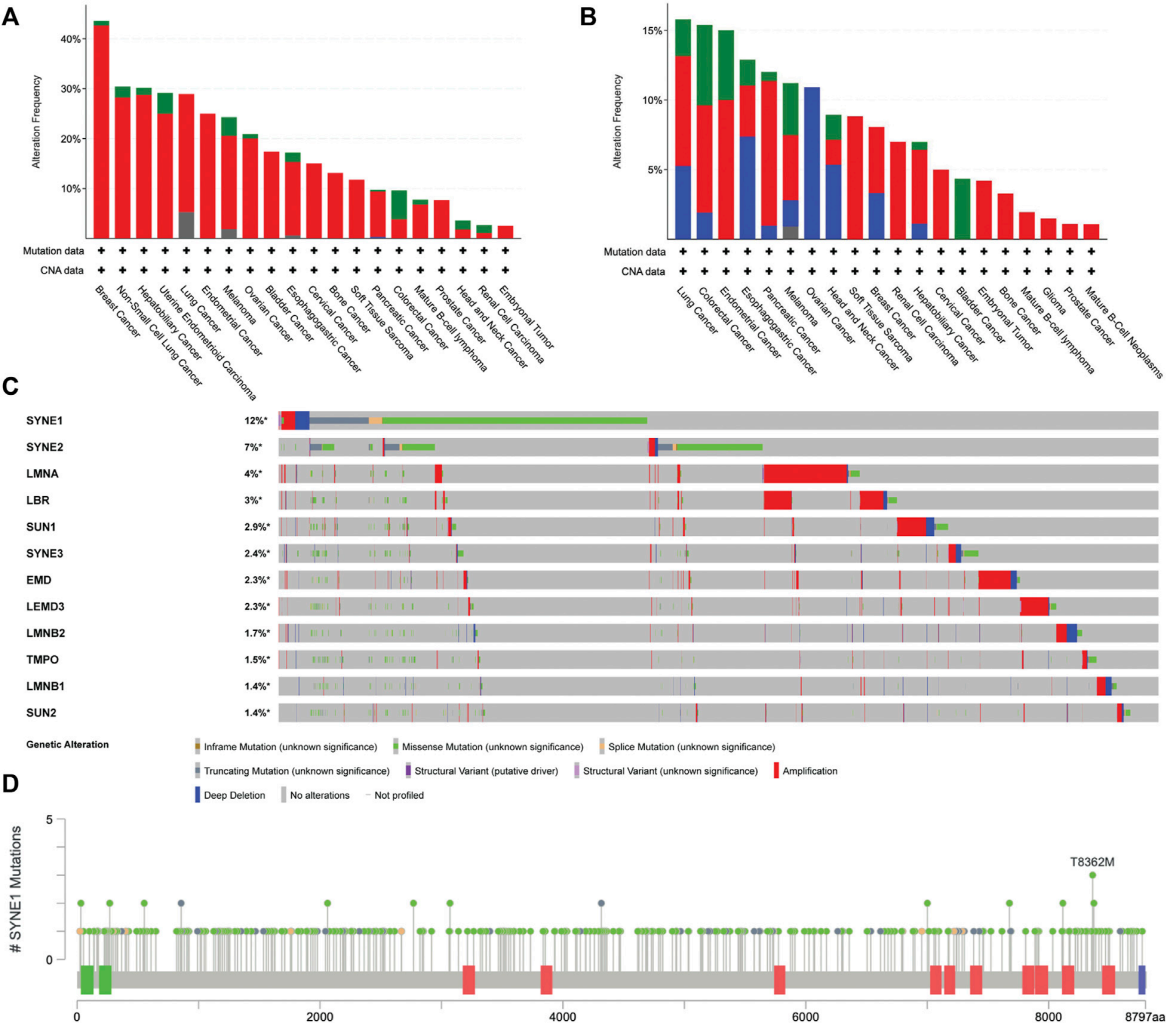
Analysis of genetic alterations in nuclear envelope genes in cancer patients using cBioPortal data (ICGC/TCGA Pan-Cancer Analysis of Whole Genomes Consortium 2020) CNAs in **(A)** LMNA **(B)** LMNB (B1 and B2) **(C)** Oncoprint of 10 nuclear envelope genes across 10967 cancer patient samples arranged in descending order based on the frequency of missense mutations **(D)** Localization and frequency of SYNE1 mutations in cancer patients using lollipop plot of cBioPortal (Cerami et al., 2012; Gao et al., 2013).

Assessing alterations in chromosome numbers is a key method for cancer diagnostics. Tumorigenesis often involves the progressive acquisition of genetic alterations in specific genes. Copy Number Alterations (CNAs) have been identified and characterized across a wide range of cancer types using high-density single nucleotide polymorphism (SNP) arrays (Chen et al., 2013). Determining therapeutic strategies in the case of deep deletions becomes especially difficult owing to the absence of the gene in the cancer cell genome, with its functional redundancy prepending an added layer of complexity. Teasing apart whether CNAs serve as drivers of cancers or silent passengers of its effects remains an area of active investigation. Here, we review the most recent advances in our understanding of the interplay between CNAs and cancer progression through data curated from cBioportal Figure 3 (Cerami et al., 2012; Gao et al., 2013).

#### 2.3.1 Amplifications

It is well established that CIN and aneuploidy involving whole chromosomal and focal amplifications and deletions in the genome are defining features of cancer initiation and progression (Watanabe et al., 2001; Zhou et al., 2002; Kops et al., 2004). Interestingly, genes that encode for lamins are strikingly amplified as compared to the other nuclear envelope genes, implying that the very mechanisms that protect genomic integrity aberrate in cancers. TCGA analyses of nuclear envelope genes across patient samples revealed CNAs of the LMNA-coding sequence in ∼13% of cancers. Breast cancer patient samples show the maximum extent of CNAs in LMNA in ∼40% of the 211 patient samples (Figure 3A). However, the extent to which gene amplifications in LMNA correlate with changes in its transcript level remains unclear. An intriguing possibility is that transcriptional deregulation of lamin A/C potentially impacts expression levels of B-type lamins or LINC genes as a consequence of copy number amplifications of lamins and the stability of their interacting partners in a cell-type-specific manner.

#### 2.3.2 Deletions

Interestingly, the LMNB1 gene shows a significant number of deletions across cancers. It is unique that LMNB1 and LMNB2 genes showed only deep deletions in ovarian cancers (Figures 3B,C). In ovarian cancer cells (HO-8910PM), decreased expression levels of lamin A/C correlate with increased cell migration and poor prognosis (Wang et al., 2019). Moreover, the lamin A:B stoichiometric ratio shows a dominance of A-type lamins in stiffer cartilaginous tissues, while B-type lamins are more prominent in softer tissues such as the brain (Swift et al., 2013). Nevertheless, if lamin A:B stoichiometry does modulate the malignant potential of cancer cells, the extent of complementation and the mechanisms that regulate the altered sub-interactome of the A- and B-type lamins remain to be uncovered.

#### 2.3.3 Mutations in nuclear envelope genes—Nesprin (SYNE1)

We examined the status of mutations in genes that encode the nuclear envelope proteins across cancers using cBioPortal. This analysis revealed recurrent mutations in the Nesprin-1 gene—SYNE1 (Figure 3D). Markedly, the SYNE1 gene accumulates the highest number of missense mutations (271), followed by truncating (41) and splice mutations (12). A mutation in exon 33 of the SYNE1 gene modifies a conserved residue in spectrin repeat 11, showing aberrant mitotic phenotypes such as altered distance between the centrosome and the nucleus, potentially contributing to CIN in human hepatoma-derived Huh7 cells (Sur-Erdem et al., 2020). Furthermore, mutation analysis of SYNE1 revealed frequent missense mutations of T8362M across three different cancers—medulloblastoma, pancreatic adenocarcinoma, and ovarian epithelial tumor. However, the physiological significance of these mutations remains to be elucidated. Do mutations in SYNE1 destabilize or hyperactivate mechanochemical signals into the nucleus and chromatin as a consequence of its altered interaction with LINC complex factors and actin? This could further contribute to increased communication with the microenvironment and the consequent proliferation of cancer cells.

## 3 Chromatin organizers in carcinogenesis

The genome is a highly dynamic collection of genes, their regulators, and massive stretches of DNA whose function is yet to be discovered. Maintenance of genome organization involves the concerted function of numerous proteins required for the regulation of chromatin organization and gene expression. The aberrant function of chromatin organizers is associated with cancers (Table 1). Here, we examine the contribution of major chromatin organizers, namely CTCF, cohesins, and condensins, to cancer progression.

**TABLE 1:** Role of chromatin organizers in cancers.

Chromatin organizer	Gene	Cancer	Effect of dysregulation of gene	Reference
Cohesin	STAG2	Glioblastoma	Mutation in STAG2 leads to aneuploidy while its rescue enhances the chromosomal stability	Solomon et al. (2011)
	RAD21	Breast	Overexpression of RAD21 in MDA-MB-231 cells leads to poor prognosis and chemoresistance, while its knockdown reduces chemoresistance	Xu et al. (2011)
Condensin	NCAPH	Colorectal	In HCT116, NCAPH depletion decreases cell migration, arrests the cells in G2/M, and enhances apoptosis	Yin et al. (2017)
	NCAPG	Liver	NCAPG has a pro-proliferative effect in adenocarcinoma patients	Zhang et al. (2022)
	NCAPH	Prostate	Upregulation of NCAPH in prostate cancers promotes cell proliferation and helps in bypassing replication checkpoints, which might hinder cancer progression	Kim et al. (2019)
CTCF		Breast	CTCF and EGR1 reduce cell migration in TNBC cell line MDA-MB-231 by inducing the expression of Nm23-H1	Wong et al. (2021)

Mutations in genes that encode for chromatin organizers are implicated in carcinogenesis and impact chromosome organization, stability and transcriptional regulation.

### 3.1 CTCF

CCCTC-binding factor (CTCF) is a conserved zinc-finger protein that functions as a chromatin organizer and transcription factor. In association with cohesin, CTCF regulates the organization of gene loci and alternative splicing primed by its sequence-specific binding to CTCF sites. In addition, CTCF functions as an insulator to restrict the expansion of repressive marks (Dixon et al., 2012; Holwerda and de Laat, 2013). The human genome has ∼55,000–65,000 CTCF binding sites, amongst which around ∼5,000 are highly conserved across species (Yusufzai et al., 2004; Chen et al., 2012; Holwerda and de Laat, 2013), though CTCF occupancy remains tissue- and cancer-specific (Hanssen et al., 2017; Debaugny and Skok, 2020). As per the loop extrusion model for TAD formation, the cohesin complex moves along the chromatin, establishing a loop until it encounters an oriented CTCF dimer. Consequently, further advancement of the cohesin complex is aborted, thus demarcating TAD boundaries enriched in CTCF binding sites (Sanborn et al., 2015; Fudenberg et al., 2016). CTCF also prevents non-specific promoter-enhancer interaction by delimiting the loop size, thus augmenting enhancer-blocking mechanisms (Amankwaa et al., 2022). The Yin Yang 1 (YY1) protein interacts with cohesin and is enriched near enhancer-promoter contact sites, thus assisting CTCF in augmenting enhancer-promoter interaction (Figure 1) (Weintraub et al., 2017).

Aberrant expression or occupancy of CTCF is associated with breast, lung, endometrial, gastrointestinal, prostate, and skin cancers (Eldholm et al., 2014; Kemp et al., 2014; Poulos et al., 2016; Guo et al., 2018; Höflmayer et al., 2020). Of note, multiple mutations map to the DNA-binding zinc finger domain of CTCF across cancers (Bailey et al., 2021). CTCF binding to its target sites is sensitive to their methylation states. For instance, hypermethylation of CTCF binding sites shows a loss of insulation in isocitrate dehydrogenase (IDH) mutant gliomas (Flavahan et al., 2016). This further leads to the ectopic interaction of the IDH enhancer with PDGFRA (platelet-derived growth factor receptor alpha), leading to its constitutive expression and the development of gliomas (Figure 1). However, not all cancer-specific mutations in CTCF affect its binding. For instance, stop codon mutations in its N- and C-terminals, as well as in the zinc finger domain, may exhibit a dominant-negative effect by hindering interactions with functionally important cofactors, thus impeding CTCF function (Debaugny and Skok, 2020).

Analyzing patient data sets from TCGA reveals frequent loss of the CTCF gene in breast and prostate cancer patients, correlating with hypermethylation of CpG islands and hypomethylation of other parts of the genome. CTCF depletion in a prostate cancer cell line, HPECE6/E7, shows hypermethylation of CTCF binding sites, further downregulating respective gene expression (Damaschke et al., 2020). This indicates that CTCF binding to its target sites prevents CpG hypermethylation and safeguards chromatin architecture, not just by organizing the chromatin but also by maintaining it. This study further reveals that drug-induced hypomethylation using 5-aza-2 deoxycytidine (5dAza) rescued chromatin organization, reaffirming the importance of CTCF and its binding sites in cancers. However, 5dAza interacts with a wide range of targets and fails to act precisely on distinct TADs, thus raising the question of specificity in cancer therapies.

CTCF functions as a double-edged sword, acting both as an oncogene as well as a tumor suppressor in a cancer subtype-specific manner. Ovarian cancers exemplify the oncogenic potential of CTCF, where metastatic lesions display elevated CTCF expression. Further, the depletion of CTCF in ovarian cancer cell lines (SKOV3 and A2780) decreased cell migration by consistently downregulating three metastasis-associated genes, including CTBP1, SRC, and SERPINE (Zhao et al., 2017). In contrast, CTCF positively regulates the expression of the metastatic suppressor, Nm23-H1, in breast cancers. Studies in the highly invasive MDA-MB-231 and the less invasive MCF-7 cells show that CTCF-dependent Nm23-H1 levels inversely correlate with cancer aggressiveness (Wong et al., 2021). The mechanism by which CTCF functions in a cancer-specific manner remains poorly understood.

Overall, changes in genome organization due to altered levels or aberrant recruitment of chromatin organizers contribute to cancer progression However, experiments performed in cell culture models need to be complemented with insights from animal models and patient-derived tumor samples. Since adherent cell culture studies are usually performed on a monolayer of cells, these approaches do not mimic the tumor microenvironment, discounting factors such as nutrient accessibility, barrier tissue formation, and variation in drug response, among others. What effects chemotherapeutic agents have on TAD organization and gene expression *in vivo* remains an area of active study. Moreover, it is intriguing that environmental factors, such as diet and social interaction, also impinge on CTCF function and, therefore, chromatin organization and function (Davis et al., 2022; Wang R et al., 2022)—an interesting finding, given that extraneous environmental factors considerably contribute to an increase in the incidence of cancers.

### 3.2 Cohesin

Cohesins are multi-protein complexes essential for mitosis and meiosis, conserved from yeast to humans. The canonical function of cohesins is to clasp sister chromatids together during the metaphase-to-anaphase transition. Apart from the aforementioned function, cohesin plays a vital role in maintaining inter-TAD and intra-TAD boundaries by looping chromatin in the interphase nucleus, allowing for regulated inter- and intra-TAD interactions (Matthews and Waxman, 2018; Barrington et al., 2019). This promotes enhancer-promoter contacts in a cell type-specific manner.

Through genome-wide sequencing, it is now apparent that cohesin accumulates a number of mutations in the coding region that alter the way it binds the chromatin and promotes aberrant genomic contacts leading to anomalous expression of various genes. Depletion of RAD21, a component of the cohesin complex, promotes enhanced expression of mesenchymal genes such as ITGA5 and TGF-B1 by altering the intrachromosomal chromatin contacts and creating active transcriptional units (Yun et al., 2016). Recent exome sequencing revealed that the STAG2 protein of cohesin is frequently mutated in cancers (Lawrence et al., 2014). It is interesting to note that STAG2 is involved in promoting regulated chromosomal contacts, the depletion of which enhances the loop extrusion and promotes aberrant genomic contacts (Adane et al., 2021; Richart et al., 2021). It remains unclear how the mutated STAG2 functions in cancer, elucidation of which might uncover a new therapeutic candidate. Moreover, the loss of cohesin function in cancers leads to increased replication stress and genomic instability (Leylek et al., 2020; Minchell et al., 2020). We surmise that cohesin mutations enhance genomic instability, facilitate clonal expansion, or enhance tumorigenic potential, eventually leading to cohesin loss of function in the clonal population. However, various lines of evidence suggest that mutations in the cohesin genes contribute to cancer initiation and progression by disrupting chromosome organization and transcriptional regulation (Table 1) (Leeke et al., 2014; Kojic et al., 2018; Antony et al., 2021).

### 3.3 Condensin

Like cohesins, condensins are multi-protein complexes required for chromosome assembly, condensation, and segregation during mitosis and meiosis. While cohesin clasps the sister chromatids together, condensin facilitates mitotic chromosome compaction by uniting the two distant portions of a single chromatid. Condensin isoforms have conserved structural maintenance of chromosome (SMC) proteins, SMC2 and SMC4, but differ in their non-SMC components. Interestingly, decreased condensin expression triggers CIN, consequently driving colorectal cancer progression (Baergen et al., 2019). In addition, mutations in the C-terminal residues R551 and S556 of CAPH2, a condensin II subunit, lead to genomic instability in the human retinal pigment epithelial (RPE1) cell line (Weyburne and Bosco, 2021). Another line of evidence shows the involvement of the condensin complex in maintaining chromosomal stability *via* its recruitment to the pericentromeric regions. The binding of cell cycle regulators pRB and E2F1 to the pericentromeric regions cause replication stress. Studies reveal that these factors recruit condensin II to form a complex in the pericentromeric chromatin, thus regulating replication fidelity and cell ploidy (Coschi et al., 2014). This agrees with an increase in the γH2A.X marker at the pericentromeric region, accompanied by enhanced repeat instability, on depletion of condensin (Samoshkin et al., 2012). However, the precise function of condensin II and the mechanistic basis of its safeguarding function against replicative stress remains to be deciphered.

Apart from chromatin compaction, condensins also play moonlighting roles that include facilitating enhancer RNA transcription and enhancer-promoter looping in condensin-bound ERα (Estrogen Receptor α)-sensitive enhancers in breast cancers by recruiting p300 and RIP140 (Li et al., 2015). Immunoprecipitation of condensins followed by mass spectroscopy or Rapid immunoprecipitation mass spectrometry of endogenous proteins (RIME) during dynamic processes such as cell transformation may reveal other non-canonical functions (Mohammed et al., 2016). Considering the limited number of therapeutic approaches available to combat triple-negative breast cancers (TNBC), it is encouraging that the knockdown of condensin I complex protein NCAPD2 curtailed cell proliferation and invasion. These lines of evidence implicate NCAPD2 expression as a prognostic marker of TNBC patients suggesting a potential therapeutic candidate (Zhang Y et al., 2020). An in-depth biochemical and molecular characterization assumes significance as condensins emerge as potential therapeutic targets for human cancers (Wang et al., 2018).

## 4 Impact of non-canonical histones and oncohistones on chromatin organization in cancers

Non-canonical histone variants occasionally replace canonical histones in the genome, often serving two main purposes. First, histone variants are dynamically incorporated throughout the interphase with the regular nucleosomal turnover of canonical histones to sustain nucleosomal stability. Secondly, additional regulatory domains, interactors, and PTMs in non-canonical histones offer supplementary mechanisms for the control of epigenetic regulation. Since cancers are characterized by large-scale remodeling of their epigenetic landscape, canonical histones in cancers are occasionally interchanged with histone variants (Vardabasso et al., 2015). Structurally, histones are composed of amino- and carboxy-terminal tails and a globular histone fold domain (HFD). Specific mutations in histone genes tend to confer oncogenic properties to cells, and these mutant histones are referred to as oncohistones (Mohammad and Helin, 2017). Mutations occur both in the tail and globular domains, with different consequences. While tail domain mutants cause a global loss of both active and inactive histone marks, the globular domain destabilizes the nucleosome. Here, we review the functional diversity and regulatory mechanisms involved in genome organization by some non-canonical and oncohistones while discussing the scope for further research in the field.

### 4.1 H3 variants

The histone variant H3.3 functions as a space-filling histone when canonical H3 is evicted from the nucleosome, thus maintaining nucleosomal stability (Ray-Gallet et al., 2011). The incorporation of histone H3.3 facilitates the enrichment of active marks on chromatin associated with dynamic histone turnovers, such as transcriptionally active promoters and enhancers of active genes (Lin et al., 2013; Ha et al., 2014). Contrastingly, histone H3.3 is also incorporated in repeat-rich and repressed telomeres, where H3.3 is incorporated into the nucleosomes and further methylated to H3.3K9me3. The H3.3K9me3 mark is vital for maintaining the integrity of constitutively heterochromatinized telomeres (Udugama et al., 2015). Specific chaperone complexes facilitate the incorporation of the histone H3.3 into different regions in the genome. In the euchromatin, H3.3 is incorporated by the HIRA complex (Shi et al., 2017; Yu et al., 2021; Yang et al., 2022), while DAXX/ATRX complexes incorporate H3.3 in the telomeric and pericentric heterochromatin (Goldberg et al., 2010; Lewis et al., 2010; Heaphy et al., 2011). Of note, DAXX/ATRX is mutated in classes of gliomas, sarcomas, and pancreatic neuroendocrine tumors and is involved in differential H3.3 deposition, thereby deregulating gene expression profiles (Heaphy et al., 2011; Yuen and Knoepfler, 2013; Ren et al., 2018). Deposition of the H3.3 variant in the telomeric regions might potentially contribute to the maintenance of cancer stem cells within tumors by activating embryonic stem cell dynamics and promoting alternative lengthening of telomeres (ALT) (Wong et al., 2009; Gulve et al., 2022). Moreover, the H3.3 recruiter ATRX is also localized to the nuclear periphery with the lamins, suggesting a possible interaction between H3.3, the telomere complex, and lamins, which collectively regulate telomere organization (Pennarun et al., 2021; Teng et al., 2021).

**FIGURE 4:**
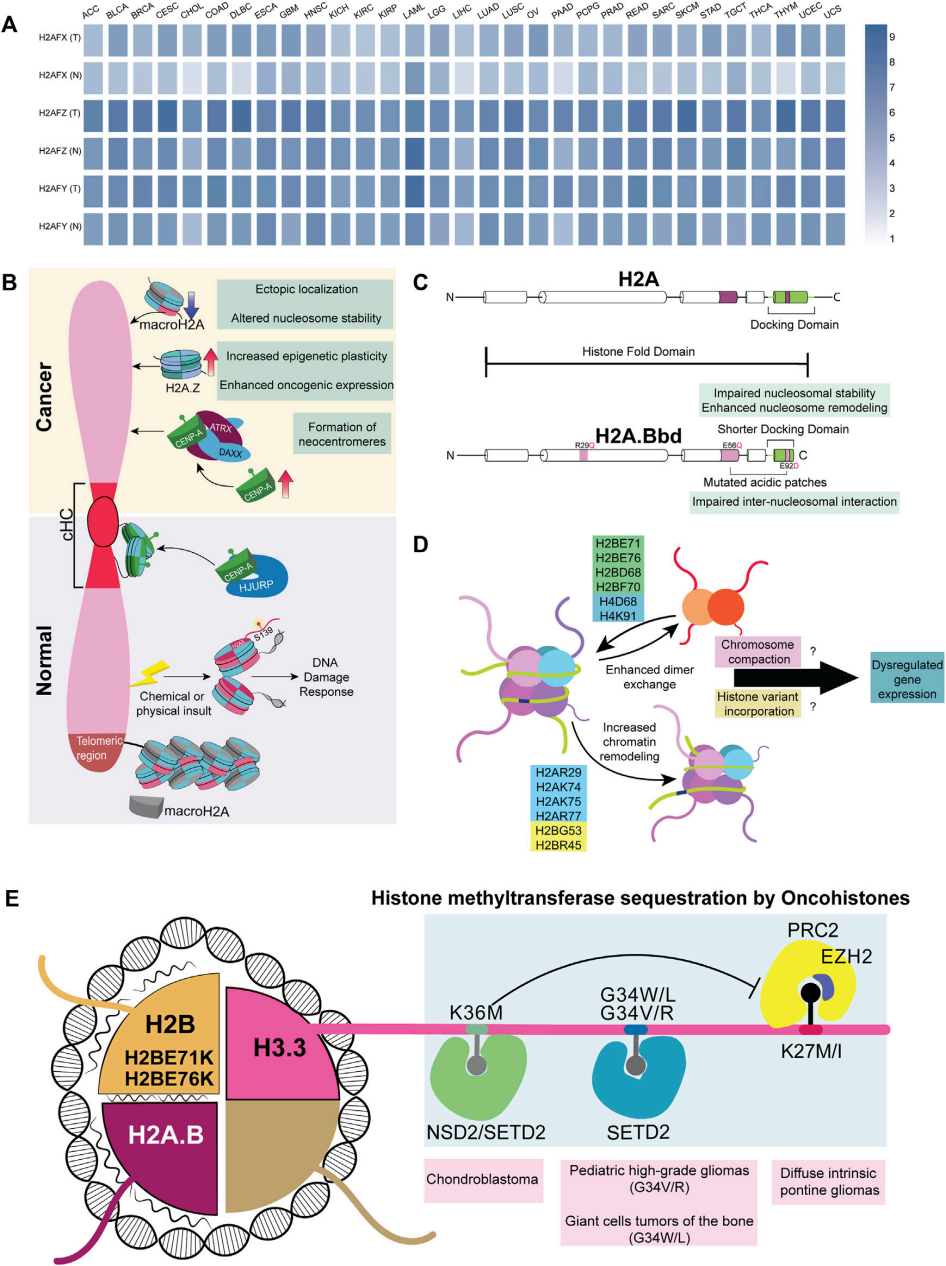
Histone variants and mutants in cancer **(A)** A heatmap representing various non-canonical histones across 31 different cancers; data curated from GEPIA2. Genes H2AFX, H2AFZ, and H2AFY code for H2A.X, H2A.Z.1, and macroH2A, respectively **(B)** Difference in the recruitment of non-canonical histones in normal versus cancer cells. cHC—Constitutive heterochromatin **(C)** Comparison between canonical H2A and non-canonical histone H2A.B **(D)** Mutations in the globular domain of core histones (H2B, H2A, and H4) to enhance dimer exchange and chromatin remodeling. This further dysregulates the expression of genes involved in differentiation **(E)** Oncohistones, mutations in the tail and globular domains are found in different cancers. Tail domain mutations sequester histone methyltransferases, while globular domain mutants destabilize the nucleosome, altering the expression of various genes.

Histone composition in the nucleosome, especially the incorporation of oncohistones, affects the expression of a wide range of genes. For instance, H3/H3.3K27M tail domain mutations accelerate neural stem cell self-renewal by dysregulating neural development genes in diffuse intrinsic pontine gliomas (DIPGs) (Mohammad and Helin, 2017; Larson et al., 2019; Nacev et al., 2019). H3G34V/R and H3.3G34W/L histone tail domain mutations are found in pediatric high-grade gliomas and giant cell tumors of the bone, respectively. Several *in vitro* and *in vivo* studies reveal that both the oncohistones, H3.3K27M and H3.3G34W/L/V/R, reduce H3K27me3 levels, resulting in the aberrant expression of Polycomb-group (PcG)-mediated heterochromatinized genes, though the results are more promising in *in vitro* systems (Mohammad and Helin, 2017). H3.3K27M tumors have enhanced expression of genes associated with neural development, where H3K27me3 loss released bivalent promoters from their poised state (Larson et al., 2019). Likewise, the H3.3K36M mutation, found in 90% of chondroblastomas, shows a parallel trend of decreasing H3K36 di- and tri-methylation, PTMs involved in RNA polymerase elongation (Jha and Strahl, 2014; Fang et al., 2016; Sahu and Lu, 2022). A possible mechanism is reducing methylation levels by the selective sequestration of histone methyltransferases, NSD2, SETD2, and PRC2, creating a dominant negative effect (Figure 4D). Intriguingly, these oncohistones affect multiple histone marks. For instance, the H3.3K36M mutation, despite decreasing H3K36 methylation, increases the deposition of H3K27me3 marks. This leads to the mobilization of the polycomb repressor complex 1 (PRC1) away from its target sites, resulting in aberrations of PcG-regulated heterochromatin and an altered epigenetic profile (Bjerke et al., 2013; Chan et al., 2013; Lu et al., 2016). It remains an open question as to why the tail domain mutants of H3.3 are spatially confined in a hindbrain tissue-specific manner. Surprisingly, the H3.3K27M mutant promotes CIN and induces NHEJ-mediated DNA damage response through the DNA end-processing enzyme Polynucleotide Kinase 3′-Phosphatase (PNKP) (Rondinelli et al., 2022). The rationale underlying the deposition of H3.3 mutants on stalled forks despite the presence of other canonical histones remains unclear. Although oncohistones function as discrete entities, the mechanistic basis underlying their potential regulatory crosstalk would be a tantalizing finding to unravel.

### 4.2 H2A and H2B variants

All histones exist as multiple variants that modulate gene expression, barring histone H4, which has only one variant. Histone H2A and H2B cumulatively have 15 non-canonical histone variants, out of which 11 are H2A variants—H2A.X, H2A.Z.1, H2A.Z.2.1, H2A.Z.2.2, H2A.Bbd, H2A.J, H2A.B, TH2A, H2A.P, macroH2A.1.1, macroH2A.2, and macroH2A1.2, and four are H2B variants—H2BE, H2B.S.M, TH2B, and H2B.W (Oberdoerffer and Miller, 2022), which are often dysregulated in cancers (Figure 4A). γH2A.X, a histone H2A subclass phosphorylated at S139, functions as a molecular beacon that detects DNA damage in the genome (Mah et al., 2010). Of note, the lack of H2A.X causes lethality in mice exposed to γ-irradiation, establishing its importance in maintaining genome stability (Celeste et al., 2002). Surprisingly, H2A.X is also involved in sustaining the self-renewal capacity of pluripotent stem cells (Turinetto et al., 2012). We speculate an H2A.X-dependent mechanism involved in the sustenance and regulation of cancer stem cells (Kim et al., 2012). As the guardian of the genome, the significance of H2A.X in facilitating metastasis was demonstrated when the knockdown of H2A.X induced EMT through the upregulation of transcription factors Twist1, ZEB, and SLUG (mesenchymal markers) in HCT116 colorectal cancer and MCF10A non-tumorigenic breast epithelial cell lines (Weyemi et al., 2016). This strongly suggests alternate functions of H2A.X in various aspects of gene regulation in addition to its role in the DNA damage response machinery.

Both H2A.Z and H3.3 maintain an open conformation of chromatin in nucleosome-depleted regions of the promoter for transcription factors to interact with gene promoters resulting in their transcriptional upregulation (Jin et al., 2009). Consistent with the requirement to transcribe genes, H2A.Z facilitates access to transcribing genomic regions by destabilizing the nucleosome, which is important in regulating stem-cell renewal and differentiation (Buschbeck and Hake, 2017). In cancers, canonical H2A is often replaced by its non-canonical variants H2A.Z.1.1 and H2A.Z.2. Remarkably, these isoforms are upregulated and positively correlate with resistance to chemotherapy in malignant stages of melanoma and pancreatic ductal adenocarcinoma (Vardabasso et al., 2015; Ávila-López et al., 2021). Furthermore, overexpression of H2A.Z correlates with poor prognosis in estrogen receptor-positive breast cancer (Hua et al., 2008). The non-canonical histone variant, macroH2A (mH2A), has a macro-domain and is involved in the inactivation of the X chromosome in mammals (Chadwick et al., 2001). In contrast to other histones, mH2A has a stabilizing effect on the nucleosome and can mediate both gene activation and repression. Notably, the depletion of mH2A dysregulates gene expression in at least nine cancers (Zink and Hake, 2016). However, the recruitment mechanism of mH2A is yet to be completely elucidated.

H2A.Bbd, a member of the short H2A family, is a testis and brain-specific histone variant overexpressed predominantly in Diffuse Large B-cell Lymphomas (DLBCLs) (Chew et al., 2021). Interestingly, H2A.B harbors multiple H2A mutations in its sequence. These include R29Q (DNA binding site mutant) and E92L (acidic-patch mutant) (Figure 4C). Furthermore, H2A.B’s truncated C-terminal tail compromises its nucleosomal compaction and, if expressed ectopically, might cause dysregulated gene regulation (González-Romero et al., 2008; Bagert et al., 2021; Chew et al., 2021; Kohestani and Wereszczynski, 2021). From a vantage point, wild-type H2A.B has already evolved into an oncohistone with the ability to promote nucleosomal instability (Bagert et al., 2021). H2B, another histone H2 variant, has the highest number of nucleosome-destabilizing mutations in the globular domain at E71 and E76 (Nacev et al., 2019; Bagert et al., 2021).

Essentially, tail-domain mutants are well-characterized, but not limited to, H3.3. The same is true for globular domain mutations, which are better documented for histone H2 (Nacev et al., 2019). Mutation data shows that 80% of the most frequent mutations in histones occur in their globular domain (Nacev et al., 2019). Many of these mutations in the globular domain enhance chromatin remodeling and histone dimer exchange, which correlates with the altered expression of genes involved in differentiation across patients with different cancers (Bagert et al., 2021). However, the mechanism and contribution of these mutations to cancers remain largely uncharacterized. We surmise that mutations in the globular domain of histones induce nucleosomal instability, which affects chromatin compaction both during mitosis and interphase. Moreover, histones bearing mutations in their globular domains mutant histones can increase the chances of incorporating histone variants, potentially altering gene expression. The temporal preference for the incorporation of histones, both dependent on and independent of replication, adds an additional layer of complexity (Figure 4E). Interestingly, 47% of the missense mutations in histones H2A, H2B, H3, and H4 show a conversion of glutamic acid residues to lysine or glutamine (Nacev et al., 2019), suggestive of 1) altered DNA-histone interactions 2) aberrations in PTM patterns of histones owing to an increase in the number of lysine and glutamine residues. Such a contribution of novel histone PTMs to carcinogenesis remains to be elucidated.

### 4.3 CENP-A

Apart from the role of histones in regulating transcription, histones are essential for modulating DNA damage response, genome organization, and chromosome maintenance. CENP-A, a centromere-specific H3 variant, is necessary and sufficient to ensure the structural and functional organization of the centromere. Heterochromatinization at the centromere is achieved by recruiting RNAi-based DICER machinery and SUV methyltransferases (Peters et al., 2003; Folco et al., 2008). Moreover, heterochromatic regions are associated with the nuclear envelope components, contributing to an additional layer of regulation (Towbin et al., 2012; Solovei et al., 2013; Ebrahimi et al., 2018; Iglesias et al., 2020). For instance, LBR and B-type lamins are involved in the organization of the pericentric heterochromatin in the interphase nucleus (Shimi et al., 2008; Dechat et al., 2010; Lukášová et al., 2018). Centromeres and telomeres are enriched in constitutive heterochromatin marks that frequently localize to the LADs in the genome (Haaf and Schmid, 1991; Weierich et al., 2003; Raz et al., 2008; Bloom, 2014), with a subset of heterochromatic domains clustering around nucleoli as perinucleolar heterochromatin (Alcobia et al., 2000; Gdula et al., 2013).

The two fundamental functions of CENP-A include 1) centromere formation and maintenance and 2) nucleation of checkpoint assembly proteins involved in chromosomal segregation. The organization of the centromere is dynamic during the various cell cycle stages, contributing to chromatin reorganization. During the early G1 phase, CENP-A molecules form a rosette-like structure nucleated by HJURP, facilitating a 3D ring-like organization during the G1 phase (Figure 4B). During mitosis, this structure is reoriented to form a rod-like pattern (Andronov et al., 2019). Elevated levels of CENP-A form neo-centromeres due to its mislocalization along the chromosomal arms, resulting in the misorientation of microtubule fibers on the kinetochore. This leads to the abnormal segregation of chromosomes, resulting in chromosomal translocations and breakage (Barnhart et al., 2011; Sun et al., 2016). It is now established that CENP-A is recruited to DNA double-strand breaks, and its depletion leads to an impaired DDR (Zeitlin et al., 2009; Lawrence et al., 2015; Stirpe and Heun, 2022). This highlights that CENP-A is recruited to sites other than the centromeric regions, although the exact role of CENP-A in DDR remains to be characterized. The ectopic overexpression of CENP-A increases the tolerance limit to DNA insults and enhances chemoresistance (Lacoste et al., 2014). The mechanism of CENP-A recruitment to DNA breakage sites and the consequent molecular signals required for its residence and dislodgement remains to be elucidated.

## 5 Chromatin organization during senescence

Cellular senescence is a state of dormancy where the cell ceases to divide. Senescence involves shortened telomeres, increased DNA damage, stalled replication, nuclear deformities, mitochondrial dysfunction, and aberrant genome organization (Di Micco et al., 2021). After a somatic cell crosses the Hayflick limit, it reaches replicative senescence because of the end replication problem, i.e., progressive shortening of telomeres with each division cycle due to the inherent inability of DNA polymerases to correctly replicate the cytosine-rich telomere lagging-strand (Hayflick and Moorhead, 1961; Harley et al., 1990). Interestingly, this limit is often bypassed by neoplastic cells, making them immortal (Autexier and Greider, 1996). As aging progresses, the DDR machinery is compromised, predominantly increasing the predisposition to breast, prostate, lung, and colon cancers (Rossi et al., 2007; de Magalhães, 2013). Normally, these functions are tightly regulated, and the activation of oncogenes leads to aberrant replication fork progression, resulting in Oncogene-Induced Senescence (OIS) (Serrano et al., 1997; Di Micco et al., 2006; Rocha et al., 2022). It is noteworthy that cancer cells often evade OIS by altering cellular levels of p16^INK4A^, a cell cycle blocker (McLaughlin-Drubin et al., 2013).

Remarkably, extensive topological changes in chromatin compartmentalization accompany senescence, obfuscating the spatial separation between the A and B compartments. Microscopy and polymer modeling of chromatin reveals that the spatial organization of chromatin compartments is drastically altered in tumor cells. Following this finding, an additional intermediate Compartment I has been proposed that interacts with both A and B compartments in normal tissue and inclines toward the B compartment in cancerous tissue (Johnstone et al., 2020). Cells undergoing OIS show dramatic changes in chromatin architecture, with the formation of Senescence Associated Heterochromatin Foci (SAHF), characteristically enriched with facultative heterochromatic marks such as H3K9me1/2, H3K20me3, along with high-mobility group proteins and non-canonical histones such as mH2A (Narita et al., 2003; Zhang et al., 2007; Nelson et al., 2016). Chromatin polymer modeling suggests that SAHF can mobilize specific loci adjacent to heterochromatic domains in close proximity to each other in the 3D space of the nucleus, enhancing their activity in cell adhesion and cancer-related signaling (Sati et al., 2020). Alongside activating specific genes, the SAHF also affects cell proliferation by epigenetically repressing E2F target genes through the recruitment of pRB and heterochromatin factors (Narita et al., 2003). Moreover, SAHFs are not found in cells going through quiescence (Aird and Zhang, 2013). In agreement with this, the silencing of E2F-responsive elements and the formation of SAHF are characteristic of only irreversibly arrested cells, thus hinting towards an Rb-mediated mechanism for stabilizing the senescent phenotype.

The induction of senescence in cancer cells serves as a traditional therapeutic approach by targeting p53, mTOR, PI3K, and BCL-2 family proteins using senolytic agents (Lee et al., 2010; Muñoz-Espín et al., 2013; Laberge et al., 2015). However, challenges in targeting cancer cells are contributed to by tumor heterogeneity since aged patients have higher numbers of senescent cells, which can lead to fatal off-target effects by senolytic agents (Wang L et al., 2022).

## 6 Perspectives

### 6.1 Effect of lamin mutations on genome organization during cancer progression and senescence

The LADs are parts of chromatin domains that are largely in a state of repression. Surprisingly, the simultaneous loss of all lamin forms in mouse embryonic stem cells did not change the overall TAD structure but reorganized the inter- and intra-TAD interactions, further altering transcriptional output (Zheng et al., 2018). It is interesting to note that although lamins are known to organize heterochromatin proximal to the nuclear border, they also modulate the organization of transcriptionally-active euchromatin within the nuclear interior (Pascual-Reguant et al., 2018; Ikegami et al., 2020).

Ovarian cancers harbor homozygous deletion in the LMNB1 gene, while the loss of lamin A/C leads to poor prognosis and enhanced metastatic potential of cells (Capo-chichi et al., 2011). In the context of senescence, lamin A/C directly interacts with the telomeric protein TRF2, which facilitates the insertion of 3′ overhangs into telomeric DNA, resulting in T-loop formation that protects the telomere ends and slows cellular senescence in a cell-type specific manner. Mutations in LMNA, like those found in Hutchinson-Gilford progeria syndrome (HGPS), destabilize lamin A/C-TRF2 interaction, further leading to telomere loss and accelerated cellular aging (Wood et al., 2014).

**FIGURE 5:**
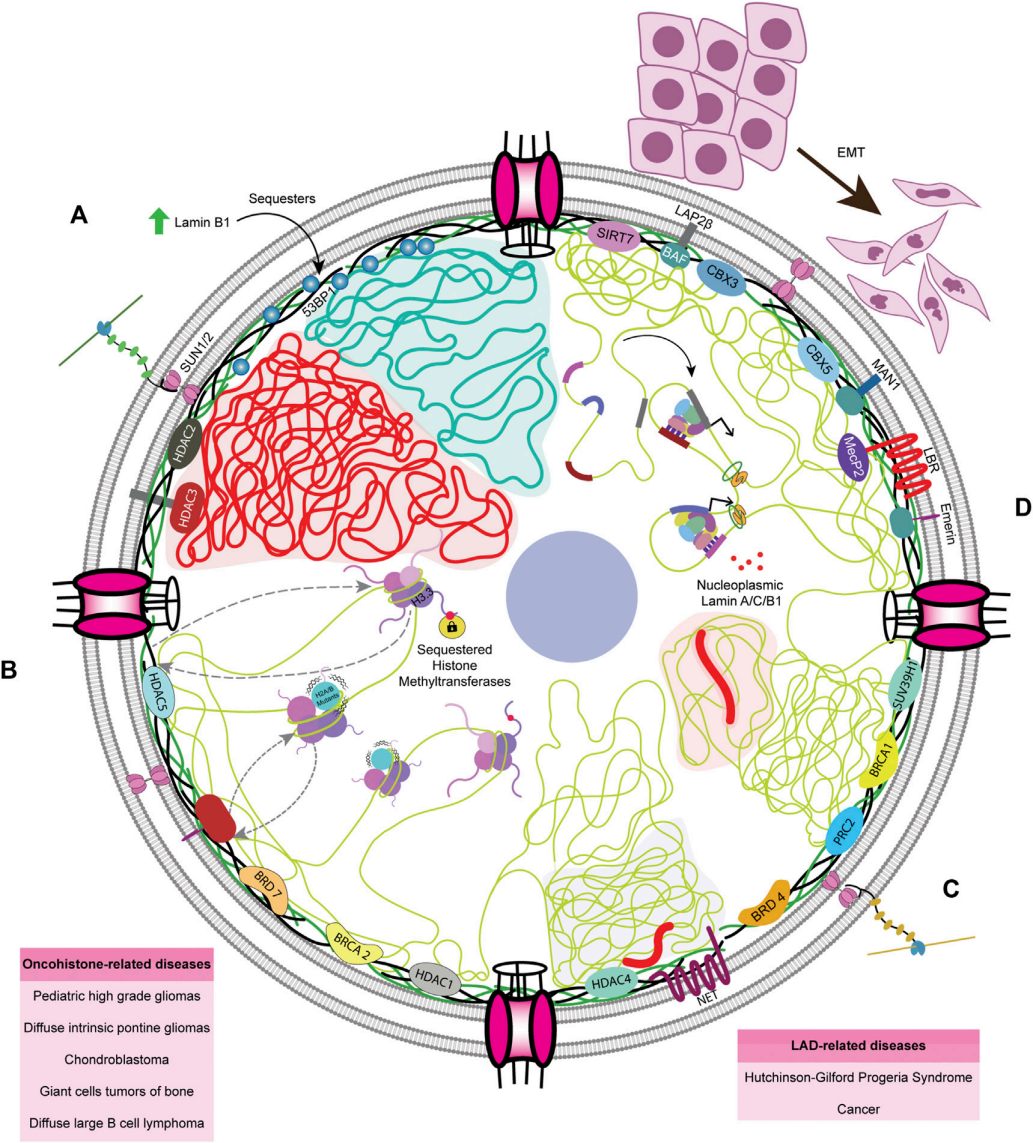
Lacunae in the field of genome organization and nuclear envelope **(A)** Lamin B1 is upregulated in most cancers, as per TCGA data, and overexpression of Lamin B1 expression sequesters 53BP1, a major orchestrator of NHEJ pathway. The precise mechanisms as to how cancer cells overcome 53BP1 sequestration is unknown **(B)** Incorporation of both tail and globular domain mutants leads to dysregulated gene expression through various mechanisms, though it remains unclear whether nuclear lamina has any role in either mitigating or worsening it. Globular domain mutations, when associated with the LADs, could be altering the spatial localization of gene loci. **(C)** The nuclear envelope maintains heterochromatin. LADs form a significant portion of the human genome and are regulated by the lamina and its associated complexes, the deregulation of which often leads to HGPS and cancer **(D)** Furthermore, in EMT and cancer progression, phosphorylated lamin A/C and B1 are associated with enhancer sequences, but the role of this association remains to be elucidated.

Additionally, TCGA data retrieved from GEPIA2 shows that most of the cancers show upregulated levels of lamin B1. It is known that lamin B1 overexpression sequesters 53BP1, a crucial mediator of the NHEJ pathway (Etourneaud et al., 2021). It remains to be elucidated how a majority of the cancers overexpressing lamin B1 manage to steer the DDR to specific NHEJ pathways (Bouwman et al., 2010) (Figure 5A). Furthermore, lamins repress the activation of mobile transposable elements that trigger chromosomal instability (Andrenacci et al., 2020). During the later stages of cellular aging, the association of lamin with transposable elements declines, coupled with the loss of heterochromatin, leading to aberrant gene expression and type-I interferon response (De Cecco et al., 2019; Cenni et al., 2020). The mechanisms by which chromatin organizers function in cancer cells will facilitate the design of specific small molecule inhibitors.

### 6.2 Interactors of LMNA during cancer progression

Lamin A regulates gene expression by interacting with various chromatin modifiers, the deregulation of which promotes cancers. For instance, lamin A directly interacts with and prevents the proteasomal degradation of SUV39H1, the writer of the H3K9me3 inactive mark (Liu et al., 2013), the dysregulation of which results in HGPS (Figure 5C). Correspondingly, the PcG proteins that deposit the H3K27me3 inactive mark interact with lamins (Cesarini et al., 2015; Marullo et al., 2016). Notably, lamin loss leads to an anomalous distribution of PcG proteins, eventually resulting in dysregulated gene expression and accelerated cancer progression. HDAC2 also plays an active role in heterochromatinization by interacting with LMNA at the nuclear periphery (Mattioli et al., 2018; Santi et al., 2020; Murray-Nerger and Cristea, 2021). However, the molecular mechanisms involving lamin-mediated regulation of inactive H3K9me3 and H3K27me3 and active H3K4me3 marks are yet to be uncovered.

In cancers, chromatin organizers harbor mutations in various domains, resulting in their deregulated activity and altered binding to chromatin or lamins. For instance, sarcomas harbor high-frequency H3.3G34R and H3.3K36M mutations that directly prevent the binding of H3K36me2/3 writer NSD1/2, thus reducing PRC2-H3K36me2 interaction and increasing H3K27me3 levels (Lu et al., 2016). The interaction between SMARCB1 and NSD1 is essential for the deposition of H3K36me2 in the genome, which is a marker for better prognosis in sarcoma. Mutated SMARCB1 is unable to bind to NSD1 but binds to PRC2, leading to an increase in H3K27me3 with poor prognosis in cancer patients (Drosos et al., 2022). Such atypical deposition of inactive histone marks dilutes their occupancy in normally-repressed genes, reorienting the genomic regions localized to the nuclear periphery. As a result, genes typically localized to the nuclear periphery, such as the mesenchymal progenitor genes in the facultative LADs, become de-repressed (Lu et al., 2016). Hence, we surmise a potential crosstalk between the nuclear lamins, chromatin regulators, and oncohistones in the initiation and sustenance of cancer progression.

### 6.3 Crosstalk between the nuclear envelope and oncohistones

The nuclear lamina is primarily associated with inactive histone marks at the nuclear periphery. However, lamins also modulate active euchromatin (Zheng et al., 2018). How the peripheral and nucleoplasmic pools of lamins engage in chromatin dynamics and impinge on the transcriptional regulation mediated by oncohistones such as H3.3K27M/I, H3.3G34W/V/L/R, H3.3K36M, or sH2A is unclear (Figure 5B). In addition, components of the nuclear envelope, namely Nups and LINC complex factors, also participate in chromatin organization. Moreover, lamins are required for chromatin organization, although their potential role in the incorporation of oncohistones by chaperones remains unclear (Figure 5B). The extent of lamin A/C phosphorylation correlates with lamin A/C levels in the DU145 prostate cancer cell line, though this remains to be verified experimentally across cancers (Kong et al., 2012). Phosphorylated lamin A/C and unphosphorylated, probably nucleoplasmic lamin B1, associate with active enhancers and transcribing genes, respectively, which contradicts the conventional function of LADs in gene repression (Guelen et al., 2008; Ikegami et al., 2020). However, the exact role of phosphorylated Lamin A/C in modulating enhancer regions remains an enigma. In addition, the association of lamin B1 with eLADs and higher expression of fibronectin, vimentin, and twist highlight the role of lamin B1 in metastasis (Pascual-Reguant et al., 2018), although its exact purpose of lamin B1 in compartment A remains to be elucidated (Figure 5D). Interestingly, lamin B1 has been shown to localize to the TAD borders of eLADs, opening the possibility of its interaction with border elements such as CTCF and cohesin.

## 7 Conclusion

In summary, it is beyond any doubt that genetic mutations, aberrations in chromatin organization, incorporation of oncohistones, deregulated transcription, and defects in nuclear envelope organization and function collectively deregulate the cellular and molecular processes of cancers. Novel therapeutic targets will be identified by leveraging high-resolution single-cell approaches, such as sc-ChIP-seq, sc-ATAC-seq, and sc-Hi-C, with high-content imaging, including high-resolution FISH. Furthermore, molecular and biochemical assays remain the mainstay for the elucidation of molecular mechanisms. Collectively, these lines of evidence reveal that aberrant genome architecture serves as a precursor and promoter of cancer initiation and progression.
